# Enhancing the antibacterial activity of antimicrobial peptide PMAP-37(F34-R) by cholesterol modification

**DOI:** 10.1186/s12917-020-02630-x

**Published:** 2020-11-02

**Authors:** Liangliang Chen, Tengfei Shen, Yongqing Liu, Jiangfei Zhou, Shuaibing Shi, Yang Wang, Zhanqin Zhao, Zhiling Yan, Chengshui Liao, Chen Wang

**Affiliations:** 1 The Key Lab of Veterinary Biological Products, Henan University of Science and Technology, Luoyang, Henan China; 2 Henan Provincial Open Laboratory of Key Disciplines in Environmental and Animal Products Safety, Henan University of Science and Technology, Luoyang, Henan China; 3 Jiaozuo Center for Animal Disease Prevention and Control, Jiaozuo, Henan China

**Keywords:** Antimicrobial peptide PMAP-37(F34-R), Cholesterol, Hydrophobicity, Antibacterial activity, Therapeutic efficacy

## Abstract

**Background:**

The problem of increasing resistance against conventional antibiotics has drawn people’s attention. Therefore, the development of novel antibacterial agents with effective and safe therapeutic effects is imminent. Antimicrobial peptides (AMPs) are considered a promising class of antibacterial agents due to their broad antibacterial spectrum.

**Results:**

In this study, on the basis of our previously studied peptide PMAP-37(F34-R), a novel antimicrobial peptide Chol-37(F34-R) was developed by N-terminal cholesterol modification to increase hydrophobicity. We observed that the N-terminal cholesterol-modified Chol-37(F34-R) showed higher antimicrobial activity than PMAP-37(F34-R) in vitro. Chol-37(F34-R) also exhibited effective anti-biofilm activity and may kill bacteria by improving the permeability of their membranes. Chol-37(F34-R) exerted high stability in different pH, salt, serum, and boiling water environments. Chol-37(F34-R) also showed no hemolytic activity and substantially low toxicity. Furthermore, Chol-37(F34-R) exhibited good potency of bacteria eradication and promoted wound healing and abscess reduction in infected mice. Meanwhile, in *S. aureus* ATCC25923-infected peritonitis model, Chol-37(F34-R) exhibited an impressive therapeutic effect by reducing the decrease in systemic bacterial burden and alleviating organ damage.

**Conclusions:**

Our findings suggested that the N-terminal cholesterol modification of PMAP-37(F34-R) could improve antibacterial activity. Chol-37(F34-R) displayed excellent bactericidal efficacy and impressive therapeutic effect in vivo. Thus, Chol-37(F34-R) may be a candidate for antimicrobial agents against microbial infection in the clinic.

## Background

The extensive use and abuse of antibiotics have constantly caused bacterial resistance [1]. Every year, a total of 700,000 human deaths occur worldwide due to bacterial resistance [2]. Antibiotic resistance results from various causes, including the inactivation of antimicrobials caused by bacterial enzymes; the low permeability of bacterial membranes; the changes in the target proteins of antimicrobials binding to bacterial membranes; the formation of bacterial biofilms. The challenge of de novo antibiotic drug discovery based on chemical entities is challenging; thus, antimicrobial peptides (AMPs) have earned considerable attention and are potential candidates in the prevention and control of bacterial infections. As a part of the innate immune system, AMPs are important contributors to the defense against invading pathogens due to their broad-spectrum activities, broad mechanisms of action, and low tendency to induce resistance [3–5]. Most natural AMPs are restricted in clinical applications due to their insufficient antimicrobial activity, high toxicity, and low stability [6–8]. Many strategies around positive polarity and hydrophobicity, including amino acid substitutions, cyclization, and fatty acid modification have been studied and adopted to overcome these disadvantages of AMPs [9, 10].

PMAP-37 is an antimicrobial peptide derived from porcine myeloma cells with 37 amino acids and α-helix structure [11]. In our previous studies, a novel antimicrobial peptide PMAP-37(F34-R) was developed via amino acid substitution on the basis of PMAP-37. PMAP-37(F34-R) displayed higher antimicrobial activity in vitro and better treatment potential in vivo compared with PMAP-37, and PMAP-37(F34-R) was proven to be a potential antibacterial agent [12]. Cholesterol is a commonly used hydrophobic compound with a planar multi-cycle unit and a flexible aliphatic chain. Given its unique structure, cholesterol has been used to modify AMPs to promote self-assembly, enhance antibacterial activity, and reduce toxicity [13–15].

In the present study, a novel N-terminal cholesterol-modified antimicrobial peptide Chol-37(F34-R) was developed to improve antimicrobial activity. The antibacterial activity, biofilm inhibition activity, biofilm eradication activity, permeability against pathogens, stability, hemolytic activity, and cytotoxicity of Chol-37(F34-R) in vitro were tested. Its potency of bacteria eradication and therapeutic effect against *S. aureus* ATCC25923-infected knife injury model, *S. aureus* ATCC25923 or *P. aeruginosa* GIM1.551-infected abscess model, and *S. aureus* ATCC25923-infected peritonitis model were investigated in vivo.

## Results

### Design and characterization of the peptides

**Fig. 1:**
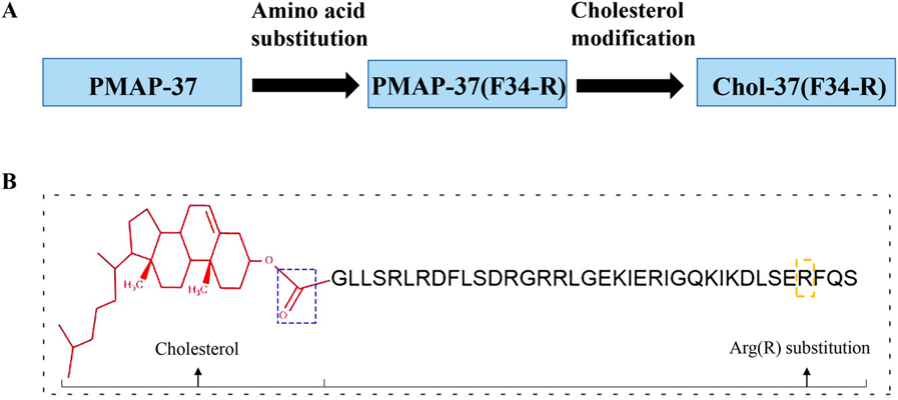
Peptides timeline and Chol-37(F34-R) molecular structure. **a** shows the timeline of peptide design from PMAP-37 to PMAP-37(F34-R) to Chol-37(F34-R). **b** shows the structure of Chol-37(F34-R). Chol-37(F34-R) was synthesized using Fmoc solid-phase peptide synthesis protocols. After removal of the Fmoc and coupling of the subsequent amino acids, the monocholesteryl ester of carbonic acid was linked to the only “-NH_2_” group of the first amino acid (Gly) of the antimicrobial peptide PMAP-37(F34-R). The blue dashed frame indicates a linker. The yellow dashed frame indicates Arg(R) substitution at position 34 of PMAP-37

**Fig. 2:**
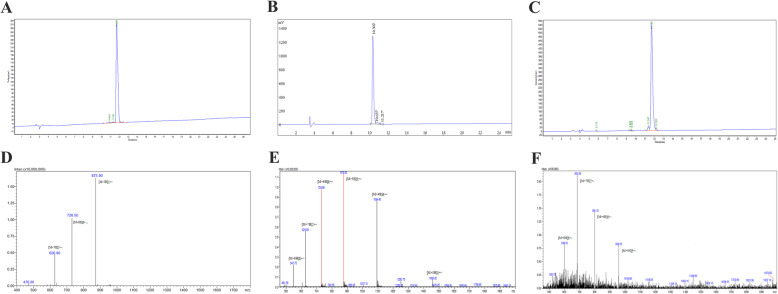
HPLC and MS analysis of Chol-37(F34-R). **a**, **b**, and **c** show the HPLC of PMAP-37, PMAP-37(F34-R), and Chol-37(F34-R), respectively. **d**, **e**, and **f** show the MS of PMAP-37, PMAP-37(F34-R), and Chol-37(F34-R), respectively

In this study, Chol-37(F34-R) was designed on the basis of the parent peptide PMAP-37(F34-R) to enhance its antimicrobial activity by increasing hydrophobicity. The N-terminus of PMAP-37(F34-R) was modified by cholesterol. Chol-37(F34-R) was synthesized using Fmoc solid-phase peptide synthesis protocols. After removal of the Fmoc and coupling of the subsequent amino acids, the monocholesteryl ester of carbonic acid was linked to the only “-NH_2_” group of the first amino acid (Gly) of the antimicrobial peptide PMAP-37(F34-R). Figure 1a shows the timeline of peptide design from PMAP-37 to PMAP-37(F34-R) to Chol-37(F34-R). Figure 1b shows the structure of Chol-37(F34-R) and delineates the design of Chol-37(F34-R) on the basis of the conjugation of saturated cholesterol to a peptide part. The sequences, biochemical parameters, high-performance liquid chromatography (HPLC), and mass spectrometry (MS) of PMAP-37, PMAP-37(F34-R), and Chol-37(F34-R) are presented in Table 1 and Fig. 2. By the identification of MS, the measured molecular weights of PMAP-37 (4364.96), PMAP-37(F34-R) (4374.07), and Chol-37(F34-R) (4786.55) were found to be nearly consistent with the theoretical molecular weights of PMAP-37 (4365.02), PMAP-37(F34-R) (4374.03), and Chol-37(F34-R) (4786.67), respectively, revealing that all peptides were successfully synthesized. The retention time of PMAP-37(F34-R) was 10.302 min, whereas that of Chol-37(F34-R) was 11.705 min. The retention time of Chol-37(F34-R) was prolonged in comparison with that of PMAP-37(F34-R), that is, the hydrophobicity increased.

**Table 1:** Amino acid sequences and biochemical parameters of PMAP-37, PMAP-37(F34-R), and Chol-37(F34-R)

Peptides	Sequence	Theoretical molecular weight	Measured molecular weight^a^	Net charge^b^	Theoretical pI^c^	T_R_ (min)^d^
PMAP-37	GLLSRLRDFLSDRGRRLGEKIERIGQKIKDLSEFFQS	4365.02	4364.96	+ 3	10.24	11.698
PMAP-37(F34-R)	GLLSRLRDFLSDRGRRLGEKIERIGQKIKDLSERFQS	4374.03	4374.07	+ 4	10.80	10.302
Chol-37(F34-R)	Chol-GLLSRLRDFLSDRGRRLGEKIERIGQKIKDLSERFQS	4786.67	4786.55	+ 3	-^e^	11.705

^a^Measured Molecular weight (MW) was measured by MS

^b^Net charge was calculated by antimicrobial peptide database. (http://aps.unmc.edu/AP/prediction/prediction_main.php)

^c^pI was calculated by antimicrobial peptide analysis tool. (https://web.expasy.org/protparam/)

^d^T_R_, retention time measured by analytical HPLC

^e^−, indicated the data not provided

### Antimicrobial activity of Chol-37(F34-R) in vitro

The Kirby-Bauer test and minimal inhibition concentration (MIC) assays were performed on *S. aureus* ATCC25923, *L. monocytogenes* CICC21634, *S. typhimurium* SL1344, and *P. aeruginosa* GIM1.551 to investigate the antimicrobial activity of Chol-37(F34-R) in vitro. Figure S1 and Table S1 show the inhibition zone results of Chol-37(F34-R) against the four bacterial strains. The area of inhibition zone represented the relative inhibition activity. The three peptides and ceftiofur sodium showed antibacterial activity against the four tested strains. Chol-37(F34-R) displayed the largest area of inhibition zone against the four tested strains, revealing the most effective bacterial inhibition. Table 2 shows the results of the MIC values of Chol-37(F34-R) against the four tested strains. The MIC values of Chol-37(F34-R) ranged from 0.0078 to 0.5 μg/mL, which were two times lower than those of PMAP-37(F34-R) against *S. aureus* ATCC25923, *S. typhimurium* SL1344, and *P. aeruginosa* GIM1.551 and four times lower than those of PMAP-37(F34-R) against *L. monocytogenes* CICC21634. In summary, the antimicrobial activity of N-terminal cholesterol-modified Chol-37(F34-R) was higher than that of PMAP-37(F34-R) in vitro.

**Table 2:** The MIC of Chol-37(F34-R) against bacteria

Bacteria Strains	MIC (μg/mL) ^a^		
	PMAP-37	PMAP-37(F34-R)	Chol-37(F34-R)
*S. aureus* ATCC25923	0.0313	0.0156*	0.0078**
*L. monocytogenes* CICC21634	4	2*	0.5**
*S. typhimurium* SL1344	4	1**	0.5**^#^
*P. aeruginosa* GIM1.551	2	1*	0.5**

^a^Minimum inhibitory concentration (MIC) was determined as the lowest concentration of peptide that inhibited bacterial growth, which was determined in three independent experiments performed in triplicate

*, *P* < 0.05 and **, *P* < 0.01, compared with PMAP-37. #, *P* < 0.05, compared with PMAP-37(F34-R)

### Biofilm inhibition and eradication activities of Chol-37(F34-R)

**Fig. 3:**
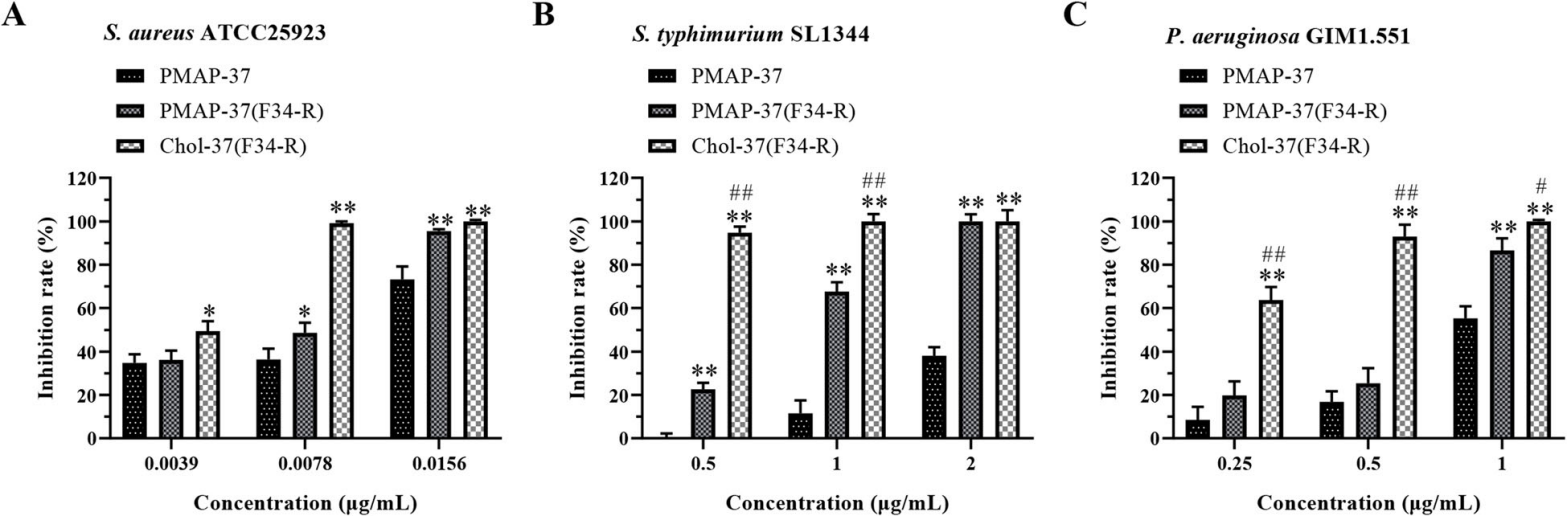
Biofilm inhibition of Chol-37(F34-R). **a**, **b**, and **c** respectively show the effect of Chol-37(F34-R) on the biofilm formation of *S. aureus* ATCC25923, *S.typhimurium* SL1344, and *P. aeruginosa* GIM1.551. The data are presented as means ± standard deviation (SD) of results (*n* = 5). *, *P* < 0.05 and **, *P* < 0.01, compared with PMAP-37. #, *P* < 0.05 and ##, *P* < 0.01, compared with PMAP-37(F34-R)

**Fig. 4:**
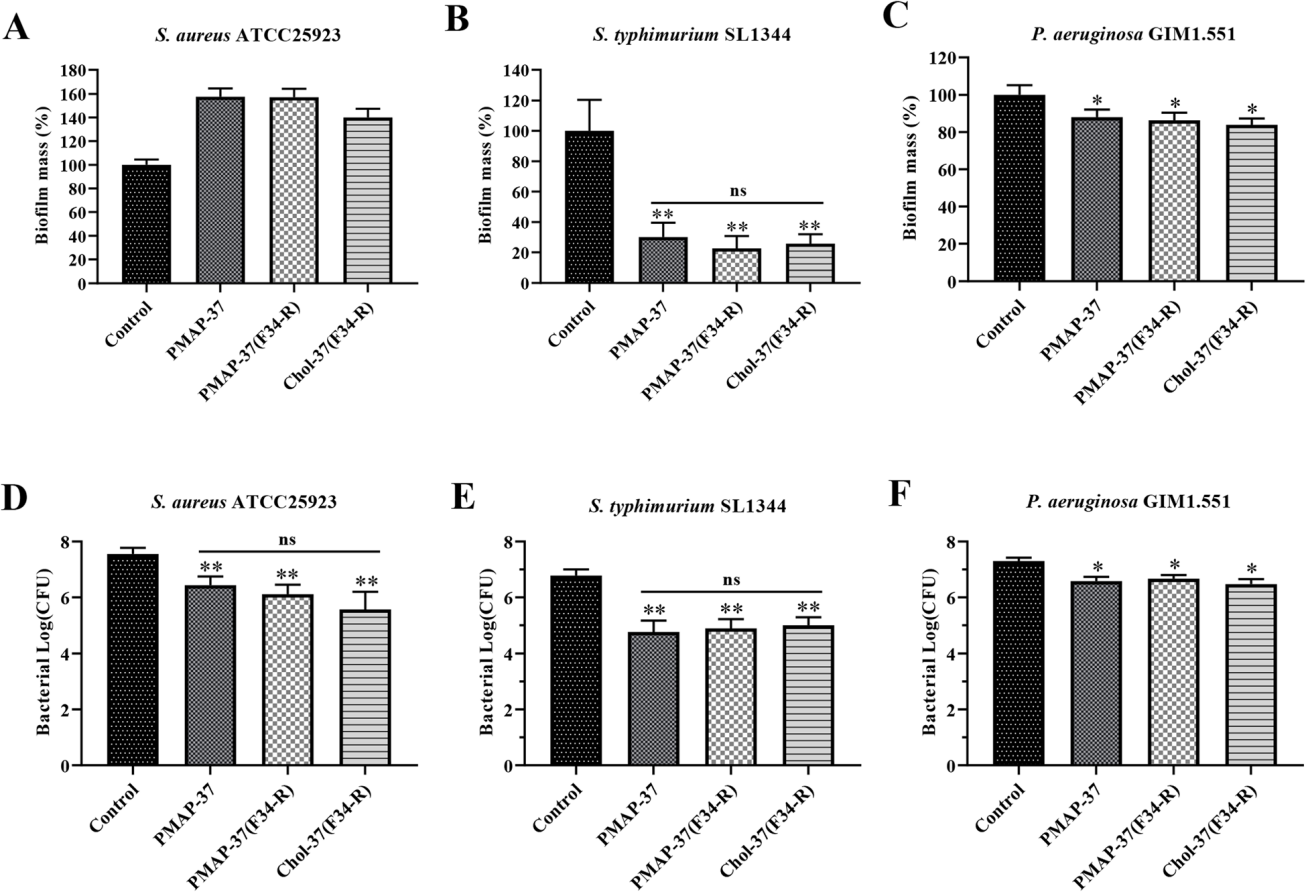
Biofilm eradication of Chol-37(F34-R). **a**, **b**, and **c** respectively show the effects of Chol-37(F34-R) on biofilm eradication of *S. aureus* ATCC25923, *S. typhimurium* SL1344, and *P. aeruginosa* GIM1.551. **d**, **e**, and **f** respectively show the killing effect of Chol-37(F34-R) against *S. aureus* ATCC25923, *S. typhimurium* SL1344, and *P. aeruginosa* GIM1.551 under the biofilm. The number of bacteria (CFU) is displayed in the form of log. The data are presented as means ± SD of results (*n* = 5). *, *P* < 0.05 and **, *P* < 0.01, compared with control. ns, nonsignificant difference

*S. aureus* ATCC25923, *S. typhimurium* SL1344, and *P. aeruginosa* GIM1.551 were tested strains to investigate biofilm inhibition and eradication capability of Chol-37(F34-R). As shown in Fig. 3, PMAP-37, PMAP-37(F34-R), and Chol-37(F34-R) dose dependently inhibited biofilm formation in the tested strains. PMAP-37(F34-R) and Chol-37(F34-R) showed a more efficient level of biofilm inhibition compared with PMAP-37. However, the Chol-37(F34-R) showed the most effective level of biofilm inhibition. As shown in Fig. 4a, b, and c, PMAP-37, PMAP-37(F34-R), and Chol-37(F34-R) exhibited the same level of biofilm eradication capability against Gram-negative bacteria *S. typhimurium* SL1344 and *P. aeruginosa* GIM1.551. However, the pre-formed biofilm of Gram-positive bacteria *S. aureus* ATCC25923 cannot be eliminated by the three peptides. All peptides resulted in death of the tested strains in the biofilm, and no significant difference was observed among the three peptides (Fig. 4d, e, and f).

### Membrane permeability of Chol-37(F34-R)

**Fig. 5:**
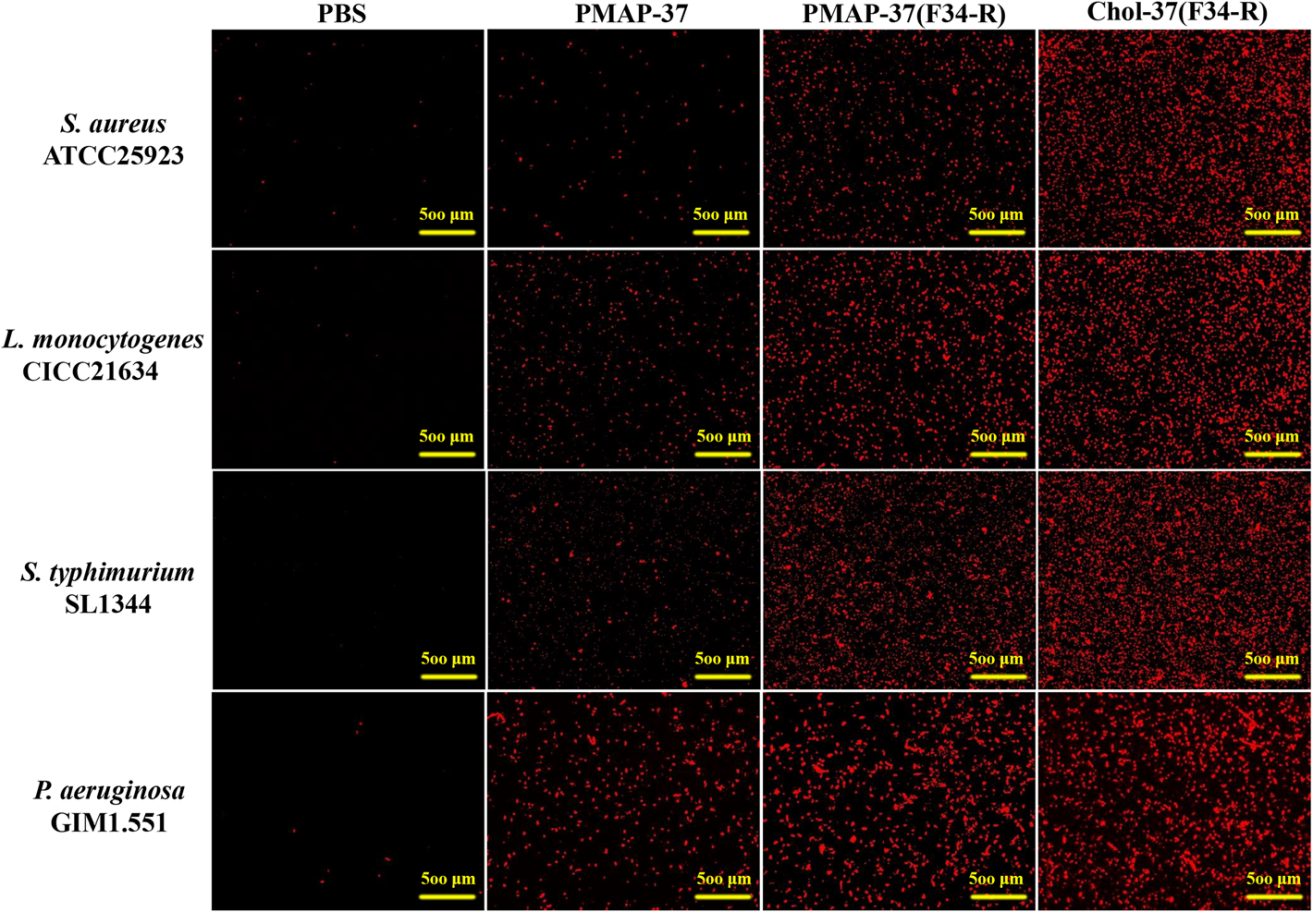
Membrane permeability against bacteria by Chol-37(F34-R). Fluorescence inverted microscopy imaging of *S. aureus* ATCC25923, *L. monocytogenes* CICC21634, *S. typhimurium* SL1344, and *P. aeruginosa* GIM1.551 after treatment with PMAP-37, PMAP-37(F34-R), and Chol-37(F34-R) for 20 min. Original magnification, × 40. The measured resolution of each image was 300 × 300 DPI and the resolution of the combined image was 300 × 300 DPI

Propidium iodide (PI) uptake assays were performed to determine the membrane permeabilizing activity of Chol-37(F34-R). As shown in Fig. 5, all PBS groups exhibited minimal or no red fluorescence against *S. aureus* ATCC25923, *L. monocytogenes* CICC21634, *S. typhimurium* SL1344, and *P. aeruginosa* GIM1.551, indicating that bacterial membranes were not damaged. By contrast, PMAP-37 groups exhibited a small amount of red fluorescence, whereas PMAP-37(F34-R) groups displayed more red fluorescence against the four tested strains. However, the largest amount of red fluorescence was shown in the Chol-37(F34-R) groups, indicating that a lot of bacterial membranes were damaged. The results indicated that the three peptides can cause damage to bacterial membranes. The permeability of the bacterial membranes of Chol-37(F34-R) was stronger than that of PMAP-37(F34-R). Thus, the results demonstrated that introduction of cholesterol strengthened the permeability of Chol-37(F34-R) toward the bacterial membranes.

### Stability of Chol-37(F34-R) exposed to pH, salt, serum, and heat

**Fig. 6:**
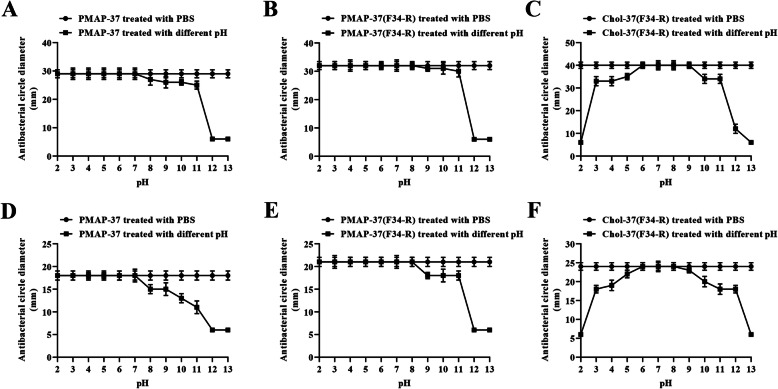
Antibacterial activity of Chol-37(F34-R) treated at different pH. pH stability test was performed using Chol-37(F34-R) treated at different pH (2–13) by disk diffusion. **a**, **b**, **c** show the pH stability curves of PMAP-37, PMAP-37(F34-R), and Chol-37(F34-R) treated with different pH solutions (2–13) with *S. aureus* ATCC25923 as the test strain, respectively. **d**, **e**, and **f** show the pH stability curves of PMAP-37, PMAP-37(F34-R), and Chol-37(F34-R) treated with different pH solutions (2–13) with *P. aeruginosa* GIM1.551 as the test strain, respectively. The data are presented as means ± SD of results (*n* = 5)

**Fig. 7:**
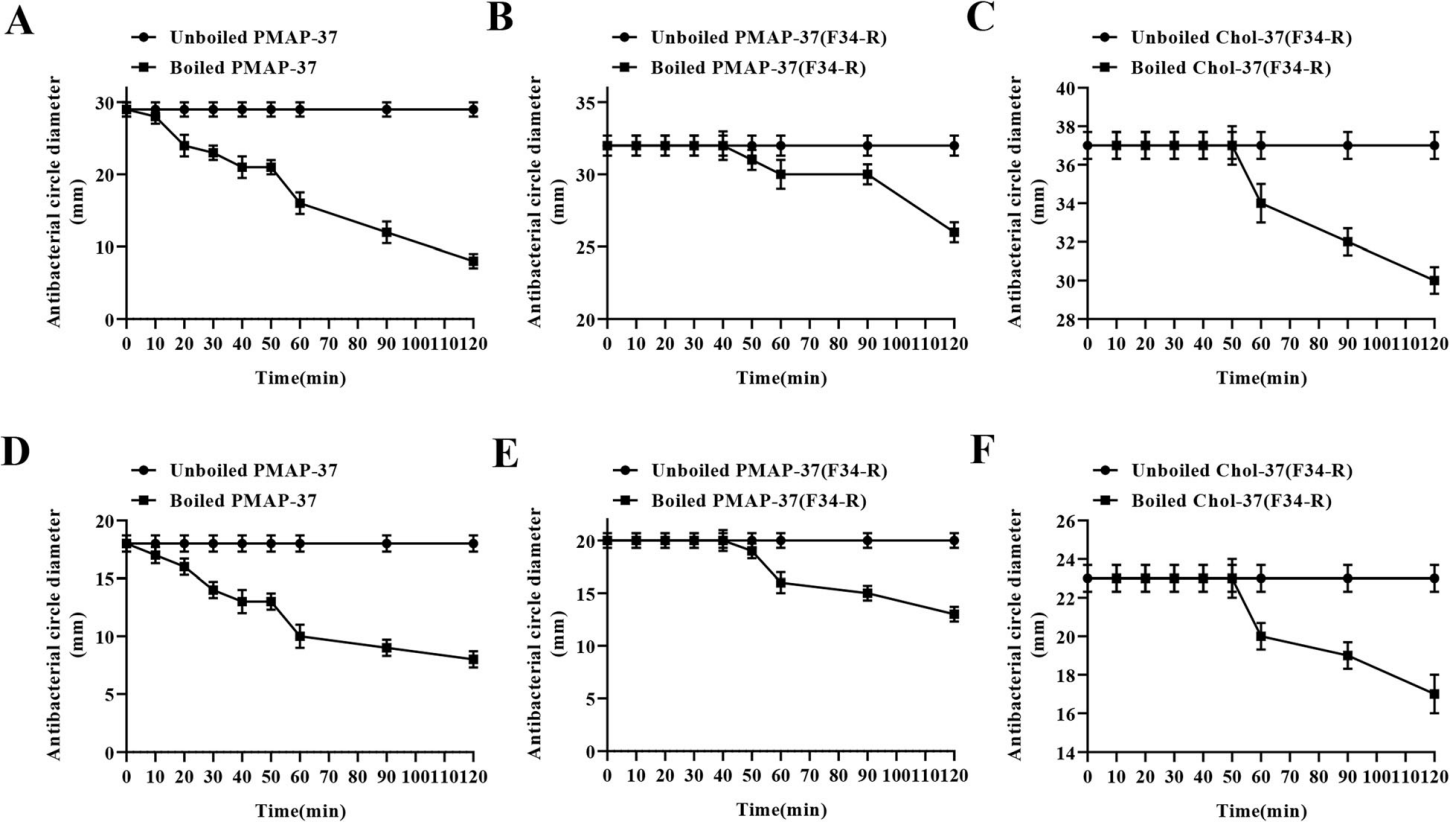
Heat-stable determination of Chol-37(F34-R) by boiling. Thermal stability test was performed using Chol-37(F34-R) boiled at different times (0, 10, 20, 30, 40, 50, 60, 90, 120 min) by disk diffusion. **a**, **b**, and **c** show the thermal stability curves of PMAP-37, PMAP-37(F34-R), and Chol-37(F34-R) with *S. aureus* ATCC25923 as the test strain, respectively. **d**, **e**, and **f** show the thermal stability curves of PMAP-37, PMAP-37(F34-R), and Chol-37(F34-R) with *P. aeruginosa* GIM1.551 as the test strain. The data are presented as means ± SD of results (*n* = 5)

Inhibition zone and MIC assays were performed to investigate the effects of pH, salt, serum, and heat on Chol-37(F34-R) activity. As shown in Fig. 6, PMAP-37(F34-R) exhibited a stable antibacterial activity against *S. aureus* ATCC25923 or *P. aeruginosa* GIM1.551 at pH 2–8. However, Chol-37(F34-R) showed a stable antibacterial activity at pH 6–9 and even exhibited certain antibacterial activity at pH 13. The results indicated that compared with PMAP-37(F34-R), Chol-37(F34-R) possessed stronger resistance to alkaline environment. Moreover, the effects of different physiological salts and serum on the antimicrobial activities of the peptides were assessed. As shown in Table S2, when NaCl, CaCl_2_, and serum were adjusted to physiologic concentrations, the MIC values of PMAP-37, PMAP-37(F34-R), and Chol-37(F34-R) against *S. aureus* ATCC25923 or *P. aeruginosa* GIM1.551 remained at the same levels as the control. The results indicated that the three peptides possessed superior resistance to NaCl, CaCl_2_, and the serum. In addition, the effects of different boiling times on the antimicrobial activities of the peptides were measured. As shown in Fig. 7, PMAP-37(F34-R) exhibited a stable antibacterial activity against *S. aureus* ATCC25923 or *P. aeruginosa* GIM1.551 at boiling time 0–40 min. However, Chol-37(F34-R) showed a stable antibacterial activity at boiling time 0–50 min, and remained in a relatively desirable active state after boiling for 120 min. The results indicated that Chol-37(F34-R) was heat-stable and had stronger heat resistance than PMAP-37(F34-R).

### Hemolysis and cytotoxicity of Chol-37(F34-R)

**Fig. 8:**
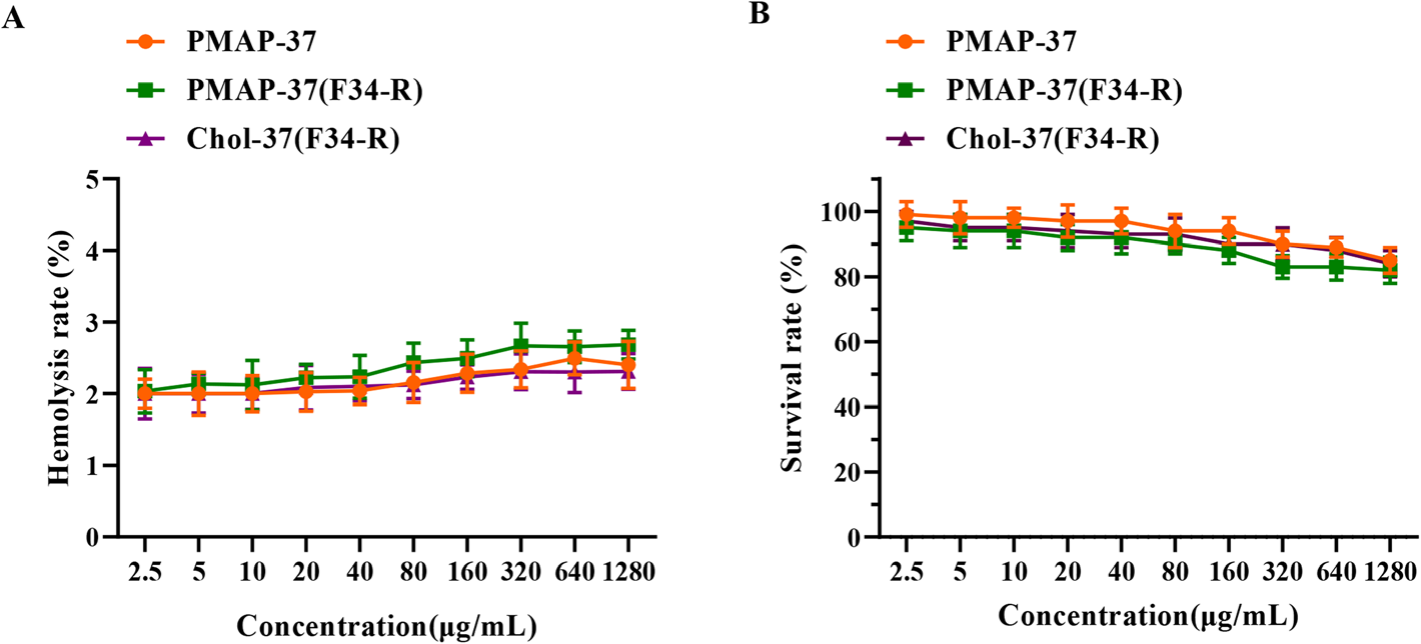
Hemolytic activity and cytotoxicity of Chol-37(F34-R). **a** shows the hemolytic activity of peptides measured by the release of hemoglobin from mouse erythrocytes. **b** shows the cytotoxicity of peptides determined by MTT assays. Mouse embryo fibroblast cell line NIH 3 T3 cells were used as target cells. The data are presented as means ± SD of results (*n* = 5)

To test the hemolytic activity of Chol-37(F34-R), the peptides (at concentrations ranging from 2.5 to 1280 μg/mL) on the cell membrane integrity of mouse erythrocytes were determined. The hemolysis rates of PMAP-37 and PMAP-37(F34-R) were less than 5% at the tested concentrations (Fig. 8a). Surprisingly, the hemolysis rate of Chol-37(F34-R) was less than 5% at 1280 μg/mL. The three peptides showed no hemolytic activity on erythrocytes, demonstrating that cholesterol modification caused no increase hemolytic activity of Chol-37(F34-R). The cytotoxicity of Chol-37(F34-R) was further evaluated against mouse embryo fibroblast cell line NIH 3 T3 cells by MTT assays. The cell viability was over 80% after treatment with PMAP-37 and PMAP-37(F34-R) (Fig. 8b). Interestingly, after Chol-37(F34-R) treatment, the cell survival rate still exceeded 80% at a concentration of 1280 μg/mL, indicating Chol-37(F34-R) had good biocompatibility toward mammalian cells and cholesterol modification caused no increase in the cytotoxicity of Chol-37(F34-R).

### Chol-37(F34-R) protects mouse against knife injury infection by *P. aeruginosa* GIM1.551

**Fig. 9:**
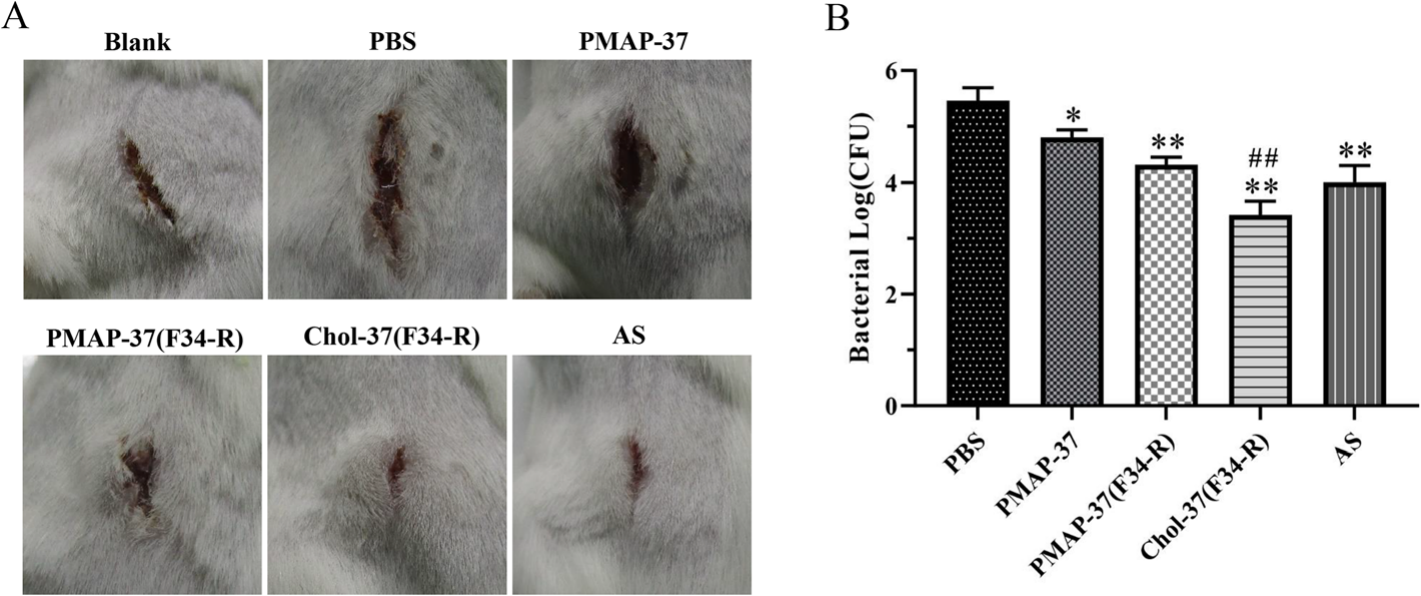
In vivo analysis of Chol-37(F34-R) against *P. aeruginosa* GIM1.551-infected mouse knife injury model. **a** shows typical photographs of wound at day 7 after different treatments. **b** shows normalized number of viable *P. aeruginosa* GIM1.551 isolated from wound sites at day 7 after different treatments. AS, ampicillin sodium. The data are presented as means ± SD of results (*n* = 5). The number of bacteria (CFU) is displayed in the form of log. *, *P* < 0.05 and **, *P* < 0.01 compared with PBS. ##, *P* < 0.01 compared with PMAP-37(F34-R)

*P. aeruginosa* GIM1.551-infected mouse knife injury model was investigated to test the therapeutic efficacy of Chol-37(F34-R) in vivo. Commercially available ampicillin sodium was examined in parallel. After 7 days of treatment, PMAP-37, PMAP-37(F34-R), Chol-37(F34-R), and ampicillin sodium groups showed smaller wound sizes than that of the PBS group (Fig. 9a). However, the Chol-37(F34-R) group showed the smallest wound size among all treatment groups. The results indicated that Chol-37(F34-R) could promote wound healing and possessed a better therapeutic effect than PMAP-37(F34-R). The colonization of *P. aeruginosa* GIM1.551 in each mouse was determined by agar plate dilution after 7 days of treatment (Fig. 9b). Compared with the PBS group, the other treatment groups showed lower bacterial content, indicating the effect of bacterium removal. Meanwhile, the bacterial content of Chol-37(F34-R) group was the smallest among all the treatment groups. The capability of Chol-37(F34-R) to remove bacteria was stronger than that of PMAP-37(F34-R) and ampicillin sodium in vivo. This finding indicated that N-terminal cholesterol modification provided Chol-37(F34-R) with stronger capability for *P. aeruginosa* GIM1.551 removal in comparison with PMAP-37(F34-R).

### Chol-37(F34-R) protects mouse against abscess infection by *S. aureus* ATCC25923 or *P. aeruginosa* GIM1.551

**Fig. 10:**
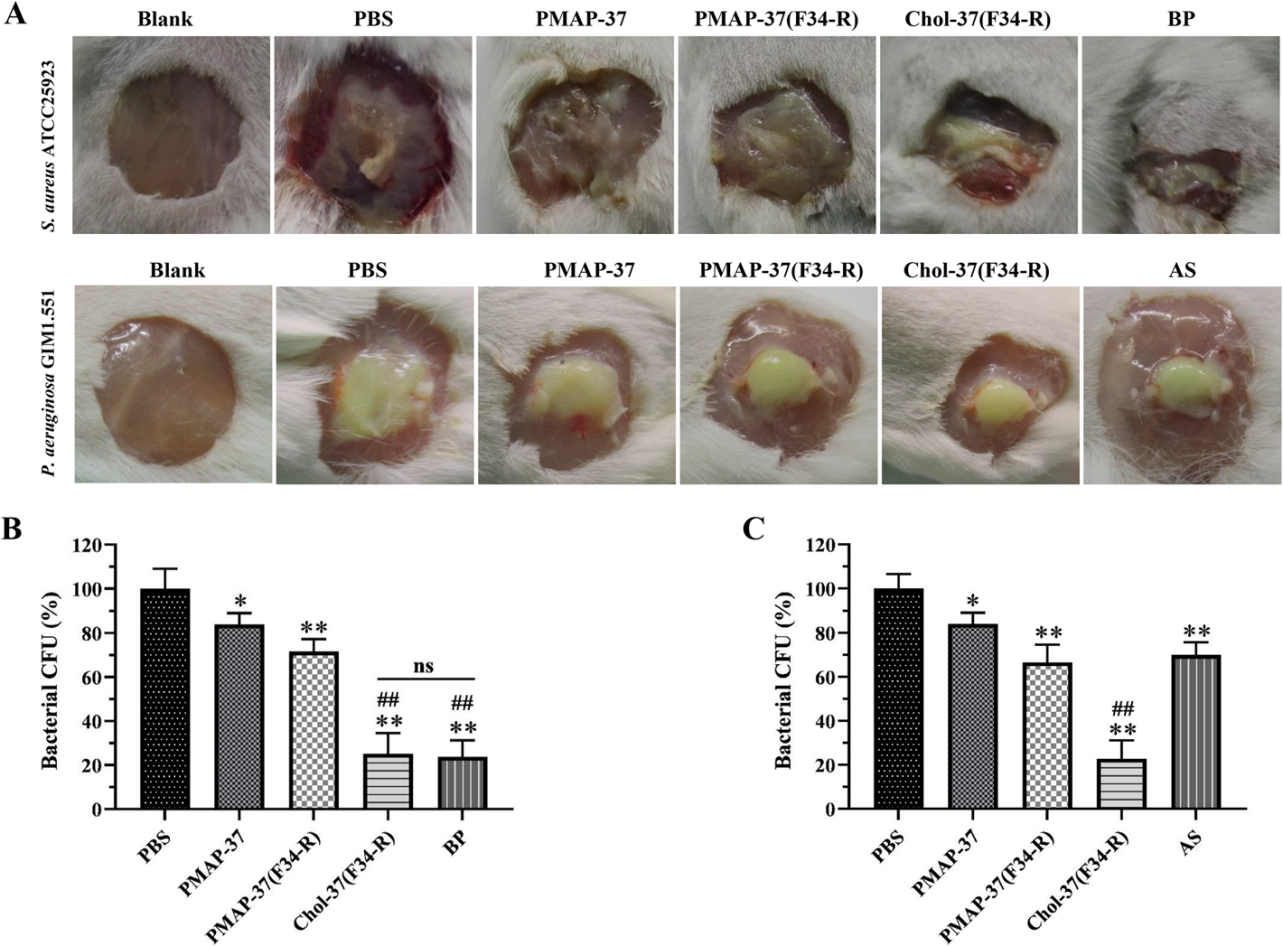
In vivo therapeutic effect of Chol-37(F34-R) against *S. aureus* ATCC25923 or *P. aeruginosa* GIM1.551-infected mouse abscess model. **a** shows typical photographs of *S. aureus* ATCC25923 or *P. aeruginosa* GIM1.551-infected abscess sites on day 3 after different treatments. **b** and **c** show the normalized number of viable *S. aureus* ATCC25923 or *P. aeruginosa* GIM1.551 isolated from abscess sites on day 3 after different treatments. BP, benzylpenicillin potassium. AS, ampicillin sodium. The data are presented as means ± SD of results (*n* = 5). The number of bacteria (CFU) in the PBS group was defined as 100%, and the other groups are displayed proportionally compared with the PBS group.*, *P* < 0.05 and **, *P* < 0.01, compared with PBS. ##, *P* < 0.01, compared with PMAP-37(F34-R). ns, nonsignificant difference

*S. aureus* ATCC25923 or *P. aeruginosa* GIM1.551-infected mouse abscess models were investigated in vivo to evaluate the potential clinical application of Chol-37(F34-R). Commercially available benzylpenicillin potassium (against *S. aureus* ATCC25923-infected mouse abscess model) and ampicillin sodium (against *P. aeruginosa* GIM1.551-infected mouse abscess model) were examined in parallel. After 3 days of treatment, compared with the PBS group, other treatment groups showed smaller abscess size (Fig. 10a). However, the abscess size of the Chol-37(F34-R) group showed smaller than that of PMAP-37(F34-R) group. Moreover, in *S. aureus* ATCC25923 model, the abscess size of the Chol-37(F34-R) group was similar to that of the benzylpenicillin potassium group. In *P. aeruginosa* GIM1.551 model, the abscess size of Chol-37(F34-R) group was less than that of the ampicillin sodium group. The results indicated that Chol-37(F34-R) had a stronger abscess removal efficiency than PMAP-37(F34-R) and ampicillin sodium and was comparable with that of benzylpenicillin potassium. The three tested peptides, benzylpenicillin potassium and ampicillin sodium reduced bacterial load in the abscess (Fig. 10b and c). In *S. aureus* ATCC25923 model, the bacterial content of the Chol-37(F34-R) group was less than that of the PMAP-37(F34-R) group and comparable with that of the benzylpenicillin potassium group. In *P. aeruginosa* GIM1.551 model, the bacterial content of Chol-37(F34-R) group was the lowest among all treatment groups. The results indicated that compared with PMAP-37(F34-R), cholesterol-modified Chol-37(F34-R) had stronger *S. aureus* ATCC25923 and *P. aeruginosa* GIM1.551 removal capability in vivo.

### Chol-37(F34-R) protects against peritonitis in mouse by reducing organ injury and bacterial burden

**Fig. 11:**
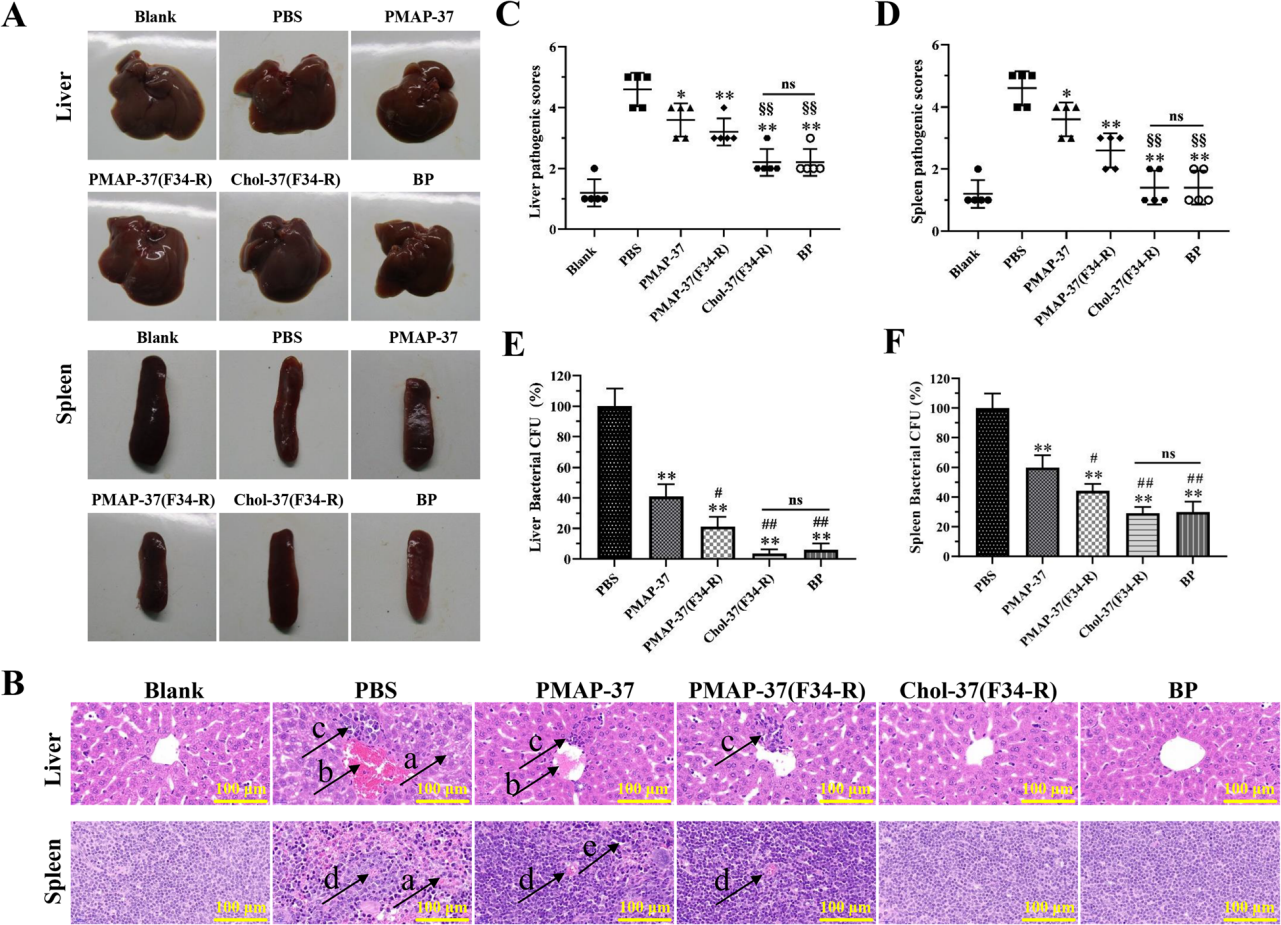
Therapeutic efficacy of Chol-37(F34-R) in *S. aureus* ATCC25923*-*infected mouse peritonitis model. **a** shows the lesions in lung and spleen of challenged mice treated with Chol-37(F34-R). **b** shows the pathological changes in the liver and spleen sections of mice challenged with *S. aureus* ATCC25923. **c** and **d** show histological scores of liver and spleen sections, respectively. Original magnification, Liver sections, × 40, spleen sections, × 40. The areas indicated by the arrows represent the type of lesion (a, necrosis; b, congestion; c, lymphocytic infiltration; d, hemorrhage; e, intumescentia). The measured resolution of each image was 300 × 300 DPI and the resolution of the combined image was 300 × 300 DPI. **e** and **f** show bacterial load of liver and spleen in mice, respectively. BP, benzylpenicillin potassium. The data are presented as means ± SD of results (*n* = 5). The number of bacteria (CFU) in the PBS group was defined as 100%, and the other groups are displayed proportionally compared with the PBS group. *, *P* < 0.05 and **, *P* < 0.01, compared with PBS. #, *P* < 0.05, compared with PMAP-37. §§, *P* < 0.01, compared with PMAP-37(F34-R). ns, nonsignificant difference

*S. aureus* ATCC25923-infected mouse peritonitis model was investigated in vivo to obtain insights into the mechanisms by which Chol-37(F34-R) effectively protects mice from infection. Commercially available benzylpenicillin potassium was examined in parallel. The liver and spleen from all tested groups were examined, and their sections were prepared for the assessment of pathological damage (Fig. 11a and b). The liver of PBS-treated mice displayed hepatocyte necrosis, formation of a large thrombus, and infiltration of inflammatory cells; splenocyte necrosis and hemorrhage were observed in the spleen. By contrast, for tissue damage, PMAP-37 treatment slightly mitigated these conditions, PMAP-37(F34-R) treatment caused partial alleviation, and treatment with Chol-37(F34-R) and benzylpenicillin potassium eliminated most of the damaged tissues. The histological scores of liver and spleen analyses also fully supported the results (Fig. 11c and d). The three tested peptides and benzylpenicillin potassium reduced the bacterial load in the liver and spleen of infected mice (Fig. 10e and f). However, Chol-37(F34-R) was remarkably more effective than PMAP-37(F34-R) and comparable with benzylpenicillin potassium in removing bacteria. Chol-37(F34-R) could protect mice from peritonitis by reducing the bacterial load and assuaging organ injury. The results indicated that cholesterol-modified Chol-37(F34-R) had stronger capability for *S. aureus* ATCC25923 removal and ability to assuage organ injury in vivo than PMAP-37(F34-R).

## Discussion

Traditional antibiotics have contributed substantially to human health and animal husbandry. However, given the undue application of antibiotics, the increase in drug-resistant bacteria poses a serious threat to public health [16–18]. Single primary target or a single mode of action of antibiotics is the main reason for the high tendency for bacterial resistance. As the first line of defense against invading pathogens, host defense peptides have evolved for billions of years. The way peptides interact with bacteria may represent the most effective model of action and the lowest propensity to induce resistance. Therefore, AMPs are an ideal template for the development of new antimicrobials. Numerous studies have been devoted to improving the cell selectivity and antibacterial activity of AMPs [9, 19]. Although the specific mechanism of action is unclear, moderate net positive charge and hydrophobicity are essential for the antimicrobial activity of AMPs [12, 20]. Net positive charge can facilitate the interactions of peptides with the negatively charged plasma membrane phospholipid molecules of the bacterial cell; after interactions, hydrophobicity could impart membrane insertion to destroy the ordered structure of the bacterial cell membrane and form pores, resulting in a loss of control over ion flows across the membrane and bacterial death [21]. Hydrophobicity is an essential parameter for the antibacterial activity of most peptides [22]. In our previous studies, PMAP-37(F34-R) was designed by using Arg (R) to replace the positions of 34 Phe (F) of PMAP-37. PMAP-37(F34-R) showed higher antibacterial activity, stronger stability, and more effective treatment effect in infected mice compared with PMAP-37 [12]. However, PMAP-37(F34-R) still has some disadvantages. The antimicrobial activity of PMAP-37(F34-R) on Gram-negative bacteria was worse than that on Gram-positive bacteria, and PMAP-37(F34-R) showed no antimicrobial activity against *E. faecium* B21 and *S. flexneri* CICC21534. Moreover, there is still a difference between the in vivo therapeutic efficacy of PMAP-37(F34-R) and that of antibiotics. Cholesterol is widely present in biological membrane phospholipids and has high hydrophobicity. Studies have shown that cholesterol modification could enhance the antibacterial activity of peptides and reduce the toxicity of peptides [14, 15]. Especially, antimicrobial peptide G_3_R_6_TAT modified by cholesterol can promote self-assembly of peptides to form nanobiological agents; it also exhibited a high therapeutic index against *S. aureus* infection in mice [13]. In addition, cholesterol modification can prolong the half-life of peptides in vivo, thereby solving a prominent problem of short half-life caused by enzyme degradation and rapid elimination in peptide therapeutics [23]. Thus, in this study, a new antimicrobial peptide Chol-37(F34-R) was designed by coupling cholesterol with the N-terminus of PMAP-37(F34-R) to increase its hydrophobicity. The antimicrobial potency of Chol-37(F34-R) was investigated in vitro and vivo.

To find new antimicrobial candidates, antimicrobial assay was used to teste the antimicrobial activity of Chol-37(F34-R). Compared with unmodified PMAP-37(F34-R), Chol-37(F34-R) showed higher antimicrobial activity against the tested strains. The amphipathicity of peptide is an important factor in antimicrobial activity, and the antimicrobial activity and selectivity of AMPs are improved by maintaining a balance of hydrophobicity and hydrophilicity [24]. The retention time of peptides on HPLC is related to hydrophobicity, and an enhancement of retention time implies an increase in hydrophobicity [25]. The conjugation of cholesterol increased the hydrophobicity of Chol-37(F34-R). Moreover, the adherent arrangement of hydrophobic and hydrophilic amino acids from PMAP-37(F34-R) were not broken by cholesterol modification. The improved antimicrobial activity of Chol-37(F34-R) may have been caused by cholesterol modification to increase hydrophobicity, thereby promoting interaction between the peptide and bacterial surface.

The development of drug resistance and chronic infections and bacterium complemented disease pathogenicity are related to biofilm production [26–28]. Thus, finding new antimicrobials that are active or specific for the prevention or treatment of bacterial biofilms is a priority. Some AMPs can prevent biofilm formation or eradicate preformed biofilm [29, 30]. In this study, compared with PMAP-37(F34-R), Chol-37(F34-R) showed more effectively inhibited biofilm formation by *S. aureus* ATCC25923, *S. typhimurium* SL1344, and *P. aeruginosa* GIM1.551. This finding was highly likely due to the killing of planktonic bacteria in the inoculum by the direct antimicrobial activity of the peptides. Chol-37(F34-R) also showed biofilm eradication but only for Gram-negative bacteria *S. typhimurium* SL1344 and *P. aeruginosa* GIM1.551 without any effect on *S. aureus* ATCC 25923. This results was likely due to lipopolysaccharide (LPS) is the major molecular component of the outer membrane of Gram-negative bacteria, and AMPs have high affinity to LPS [31]. No significant difference was observed in the biofilm eradication of PMAP-37(F34-R) and Chol-37(F34-R). This finding may be attributed to the same interaction of PMAP-37(F34-R) and Chol-37(F34-R) with the extracellular polymeric substances (EPS) of the biofilm, arousing biofilm release. Chol-37(F34-R) serve as a potential candidate for anti-biofilm agents. The low permeability of bacterial membranes is one of the causes of antibiotic resistance. Compared with PMAP-37(F34-R), Chol-37(F34-R) showed higher permeability for bacterial membranes. The results may be due to cholesterol modification, which promoted the interaction of Chol-37(F34-R) with membranes and leaded to enhanced membrane permeability and eventually bacterial killing.

The stability of AMPs is important for storage and applications [32, 33]. Different pH environments usually influence the sensitivity of bacteria to peptides [34]. Compared with PMAP-37(F34-R), the capability of Chol-37(F34-R) to maintain stable antibacterial activity in alkaline environments has improved. Moreover, the sensitivity of AMPs to physiological salts and serum has been regarded as an inevitable problem in clinical applications. Salt ions and serum reduce the antibacterial activity of AMPs [35, 36]. In this study, all the tested peptides showed superior resistance to NaCl, CaCl_2_, and serum. The cholesterol modification strategy for the N-terminus of PMAP-37(F34-R) caused no change in the resistance toward NaCl, CaCl_2_, and serum. Additionally, traditional antibiotics as feed additives are being eliminated in the feed industry, which provides a possibility for the use of AMPs as feed additives. This condition requires the strong thermal stability of AMPs. Chol-37(F34-R) exhibited stronger thermal stability than PMAP-37(F34-R). Even when boiling for 50 min, Chol-37(F34-R) retained a highly stable antibacterial activity. Therefore, N-terminal cholesterol-modified Chol-37(F34-R) may have potential as a feed additive.

Perfect biocompatibility toward mammalian cells is a vital indicators for the clinical application of AMPs. The three studied peptides have no hemolytic activity and low cytotoxicity. Cholesterol modification caused no increase in the hemolytic activity and toxicity of Chol-37(F34-R). A difference exists between the antimicrobial activities in vitro and in vivo due to the complex physiological environment in organisms. The in vivo antibacterial activity of Chol-37(F34-R) in the knife injury, abscess, and peritonitis models were evaluated. *P. aeruginosa* and *S. aureus* are the most common bacteria that cause localized and systemic infections in hospitals, and they were used to infect mice in the present study [37, 38]. In the knife injury and abscess models, compared with unmodified PMAP-37(F34-R), Chol-37(F34-R) showed better wound healing and abscess reduction and stronger capability to remove bacteria. Moreover, in the peritonitis model, Chol-37(F34-R) exhibited better therapeutic efficacy than PMAP-37(F34-R) in bacterial infection by reducing systemic bacterial burden and inflammatory organ damage in mice. This result further demonstrated that cholesterol modification of the N-terminus of PMAP-37(F34-R) is an effective strategy. These findings may be related to direct antibacterial activity in vivo; furthermore, the exact mechanism of the protective effect is unclear. In addition, several AMPs could directly or indirectly regulate immune cells and exert immunomodulatory effects, which accelerate the elimination of bacteria [15, 39]. Whether PMAP-37, PMAP-37(F34-R), and Chol-37(F34-R) is involved in immune regulation needs further research. In vivo therapeutic bacterial inhibition and injury recovery by Chol-37(F34-R) were similar to those of benzylpenicillin potassium and surpassed those of ampicillin sodium. Chol-37(F34-R) had a substantially lower possibility to develop resistance in comparison with commercially available ones, further demonstrating that Chol-37(F34-R) is a potential antibacterial agent in the treatment of bacterial infections.

## Conclusion

The present study successfully demonstrated that cholesterol modification could be a useful strategy to enhance the antimicrobial activity of PMAP-37(F34-R). Moreover, the new antimicrobial peptide Chol-37(F34-R) exhibited effective anti-biofilm activity and may kill bacteria by improving the permeability of their membranes. Chol-37(F34-R) also showed excellent stability and good biocompatibility toward mammalian cells and a remarkable therapeutic effect in *S. aureus* ATCC25923 and *P. aeruginosa* GIM1.551-induced infection in vivo. Thus, Chol-37(F34-R) holds great potential as a novel antimicrobial agent for biomedical applications.

## Methods

### Bacterial strain, antibiotic, and mice

*S. aureus* ATCC25923 was kindly provided by Bin Tang at Jilin University. *L. monocytogenes* CICC21634 was obtained from China Center of Industrial Culture Collection (CICC, China). *S. typhimurium* SL1344 and *P. aeruginosa* GIM1.551 were isolated from clinical cases and maintained in our laboratory. NIH 3 T3 cells were kindly provided by Dr. Lu at Nanjing Medical University. Thiazolyl blue tetrazolium bromide (MTT), Dulbecco’s modified eagle medium (DMEM), and dimethyl sulfoxide (DMSO) were purchased from Sijiqing Biotech (China). Ceftiofur sodium, ampicillin sodium, benzylpenicillin potassium, sodium pentobarbital, lysogenic broth (LB) medium, brain heart infusion (BHI) medium, tryptic soy broth (TSB) medium, Mueller–Hinton broth (MHB) medium, fetal bovine serum, Triton X-100, and propidium iodide (PI) were purchased from Procell Corporation (Wuhan, China). All other chemical reagents used were analytical grade. Three hundred specific pathogen-free (SPF) BALB/c mice (aged 4–6 weeks, 20 ± 2 g; 50% male and 50% female) were purchased from Henan Province Experimental Animal Center (Henan, China). All procedures performed in studies involving animals complied with the Animal Ethics Procedures and Guidelines of the People’s Republic of China.

### Peptide synthesis

PMAP-37, PMAP-37(F34-R), and Chol-37(F34-R) were synthesized by Fmoc solid-phase peptide synthesis at Shanghai Apeptide Biological Technology Co., Ltd. (Shanghai, China). All peptides were purified by HPLC to high purity (≥95%), and the synthesized peptides were identified by MS. The HPLC and MS protocol are as follows. HPLC experiments of PMAP-37 and Chol-37(F34-R) were carried out on Waters 2690 and HPLC experiment of PMAP-37(F34-R) was carried out on SHIMADZU LC-2010HT. The instruments were equipped with a Waters SinoChrom ODS-BP column (5 μm, 4.6 × 250 mm) at a flow rate of 1.0 mL/min using a gradient of 5–65% acetonitrile in water with 0.1% TFA as the mobile phase. UV detection was performed at a wavelength of 220 nm. MS experiments of PMAP-37 and Chol-37(F34-R) were carried out on SHIMADZU LCMS-2010EV and MS experiment of PMAP-37(F34-R) was carried out on Agilent LCMS-6125B. The ESI source conditions were as follow. Nebulizer gas flow: 1.5 L/min; CDL temperature: 250 °C; capillary voltage: 1500 V; T. flow: 0.2 mL/min. The mass scan was in the range of m/z 400–2000.

### Inhibition zone assays

The antibacterial activity of Chol-37(F34-R) was detected using the Kirby-Bauer diffusion method [40]. Four bacterial strains were used as experimental strains: *L. monocytogenes* CICC21634 cultured with BHI, *P. aeruginosa* GIM1.551 cultured with TSB, *S. aureus* ATCC25923, and *S. typhimurium* SL1344 cultured with LB medium. Briefly, the bacterial cells were cultured for 12 h at 37 °C in appropriate culture media, and the bacterial cell suspension was spread on the corresponding solid medium. Then, the disks containing 10 μg peptide or ceftiofur sodium were attached to the solid medium and incubated at 37 °C for 18 h. Lastly, the diameter of the inhibition zone was measured.

### Minimal inhibition concentration (MIC) assay

The MIC values of Chol-37(F34-R) were determined using the microdilution method of the Clinical and Laboratory Standards Institute [41]. Antibacterial intensity was tested in MHB against *S. aureus* ATCC25923, *L. monocytogenes* CICC21634, *S. typhimurium* SL1344, and *P. aeruginosa* GIM1.551. The tested strains were cultured for 12 h at 37 °C. Then, the bacterial inoculum concentration was adjusted to 1 × 10^6^ CFU/mL. Twofold serial dilutions covering different concentrations from 0.0039 to 256 μg/mL for each peptide were added to media containing cultures of the tested strains in 96-well plates, which were incubated at 37 °C for 18 h. The MIC values were determined by evaluating the OD_600nm_ of the cultures.

### Biofilm inhibition assay

Biofilm inhibition assay was performed as described previously to investigate the biofilm inhibition capability of Chol-37(F34-R) [42]. The assay was conducted in TSB medium supplemented with 0.5% glucose against *S. aureus* ATCC25923, TSB medium against *S. typhimurium* SL1344, and BHI medium against *P. aeruginosa* GIM1.551. Briefly, 200 μL 1 × 10^6^ CFU/mL bacterial cells was cultured at 37 °C for 24 h, in 96-well plates, with or without PMAP-37, PMAP-37(F34-R), and Chol-37(F34-R) (0.25–128 μg/mL). Planktonic bacteria were removed, and the wells containing biofilm were washed thrice with sterile PBS solution. Subsequently, 200 μL methanol (99%) was added per well, and the wells were fixed for 20 min. After aspiration, the plates were allowed to dry. The biofilm was stained with crystal violet (0.1%) for 10 min, and the excess colorant was gently eliminated by three successive washings with sterile PBS. The stain was resolubilized in 95% ethanol, and the absorbance was measured at 620 nm. Biofilm formation inhibition rate = [1 − OD_620nm_ (peptides)/OD_620nm_ (control)] × 100%.

### Biofilm eradication assay

Biofilms of *S. aureus* ATCC25923, *S. typhimurium* SL1344, and *P. aeruginosa* GIM1.551 were grown in 96-well plates by adding 200 μL bacteria (1 × 10^6^ CFU/mL) in the medium. After incubation at 37 °C for 24 h, the wells were washed thrice with PBS. The 128 μg/mL PMAP-37, PMAP-37(F34-R), and Chol-37(F34-R) samples were prepared in fresh 96-well plates using the same media. Then, 200 μL of the suspension was transferred to the plate containing biofilm. A total of 200 μL sterile medium was added to the negative control wells. The plates were incubated at 37 °C for 1 h. After incubation, the biofilm mass was determined by measuring the absorbance after applying crystal violet stain as described above. Biofilm mass %  = OD_620nm_ (peptides)/OD_620nm_ (control) × 100%. The biofilms were solubilized with 200 μL 0.1% Triton X-100 to determine the number of viable bacteria in the biofilm. Following a 10-fold serial dilution in PBS, the bacterial cells were spread on agar plates and incubated at 37 °C for 18 h and subsequently counted.

### Permeability assay

The membrane permeabilizing ability of Chol-37(F34-R) was detected using the PI uptake assay. PI was used as a fluorescent indicator to investigate the permeability of the bacterial membrane effects of Chol-37(F34-R). PI is a fluorescent dye that could enter dead bacteria and combine with DNA to emit red fluorescence, at excitation and emission wavelengths of 535 and 615 nm, respectively. The mid-logarithmic culture of *S. aureus* ATCC25923, *L. monocytogenes* CICC21634, *S. typhimurium* SL1344, and *P. aeruginosa* GIM1.551 was centrifuged at 2000 g for 5 min and resuspended in PBS (1 × 10^8^ CFU/mL). The bacteria were treated with final concentrations of PMAP-37, PMAP-37(F34-R), and Chol-37(F34-R) (0.0313 μg/mL for *S. aureus* ATCC25923, 4 μg/mL for *L. monocytogenes* CICC21634, 4 μg/mL for *S. typhimurium* SL1344, 2 μg/mL for *P. aeruginosa* GIM1.551) at 37 °C for 20 min. PBS was added to the control assay. Subsequently, an equal volume of PI (200 μg/mL) was added and the samples were stained in the dark for 10 min. The suspensions were added to the slides with cover slips for immobilization. The bacterial cells were imaged by fluorescence inverted microscope (Axio Observer A1, Zeiss). The microscopic images were captured by ZEN (2.6 version).

### pH stability assay

To investigate the effect of pH on the antibacterial activity of Chol-37(F34-R), three peptides (1 mg/mL) with different pH buffers (pH 2–13) were incubated at 37 °C for 4 h. Then, the treated peptides were tested separately in inhibition zone assays against *S. aureus* ATCC25923 or *P. aeruginosa* GIM1.551. Each standard disk contained 10 μg peptide. The inhibition zone diameters of peptides were measured.

### Salt ion and serum stability assay

The effects of salt ion and serum on the antibacterial activities of Chol-37(F34-R) were investigated by MIC assay. *S. aureus* ATCC25923 and *P. aeruginosa* GIM1.551 were used as the tested strains. The MIC values were determined at recommended doses of NaCl (8.766 g/L), CaCl2 (0.039 g/L), and fetal bovine serum (20%) [43].

### Thermal stability assay

To test the thermal stability of Chol-37(F34-R), the three peptides (1 mg/mL) were boiled for 0, 10, 20, 30, 40, 50, 60, 90, and 120 min. Then, the treated peptides were tested separately in inhibition zone assays against *S. aureus* ATCC25923 or *P. aeruginosa* GIM1.551. Each standard disk contained 10 μg peptide. The inhibition zone diameters of peptides were measured.

### Hemolytic assay

The hemolytic activity of Chol-37(F34-R) was investigated as described previously [44]. Mouse erythrocytes were diluted to a final suspension concentration of 8% (v/v). Then, 100 μL erythrocyte suspension was incubated with 100 μL peptides at different concentrations ranging from 2.5 to 1280 μg/mL. PBS and 0.2% Triton X-100 were used as negative and positive controls, respectively. After 1 h of incubation, the tested samples were centrifuged for 5 min at 1000 g. Lastly, the absorbance of the supernatant of tested samples was measured at 570 nm. The percentage of hemolytic was obtained using the following formula: Hemolysis rate = [OD_570nm_ (peptides) − OD_570nm_ (PBS)]/[OD_570nm_ (Triton X − 100) − OD_570nm_ (PBS)] × 100 % .

### Cytotoxicity assay

The cytotoxicity of Chol-37(F34-R) was assessed by MTT assays as described previously [45]. NIH 3 T3 cells were seeded in a 96-well with a density of 1× 10^4^ cells per well and incubated in 37 °C for 24 h. Afterward, the cell medium was replaced by fresh DMEM containing various concentrations of peptides (2.5–1280 μg/mL). After 24 h incubation, 20 μL MTT agent (5 mg/mL) was added to each well and incubated for 4 h. Then, the supernatant of each well was replaced with 150 μL DMSO. The absorbance was measured by a microplate reader (FlexStation III) at 570 nm.

### Therapeutic analysis of *P. aeruginosa* GIM1.551-infected mouse knife injury model

The *P. aeruginosa* strain GIM1.551 was used to build knife wound model of mouse as described elsewhere with minor modifications [46]. A total of 30 mice were randomly divided into six groups. Briefly, the fur on the back of each mouse was removed by shaving. A 1.5 cm linear knife wound was made on the back of each mouse and infected by direct seeding with bacterial suspensions (1 × 10^8^ CFU/mL, 50 μL). One day postinfection, the wounds were administered with PMAP-37 (10 μL, 1 μg/μL), PMAP-37(F34-R) (10 μL, 1 μg/μL), Chol-37(F34-R) (10 μL, 1 μg/μL), ampicillin sodium (10 μL, 1 μg/μL), or PBS (10 μL), and the treatment program was performed once per day for 7 days. The mice with no infection and treatment were used as blank control. The wounds of mice were wrapped in gauze bandages that were changed every day. Seven days after treatment, mice were euthanized with 120 mg/kg intraperitoneal (i.p.) sodium pentobarbital and their wound tissues were collected. The wound tissues were homogenized with a basic homogenizer. Following a 10-fold serial dilution in PBS, the homogenates were spread on TSB agar plates. The bacterial burden was determined after incubation at 37 °C for 18 h.

### Therapeutic analysis of *S. aureus* ATCC25923 or *P. aeruginosa* GIM1.551-infected mouse abscess model

The abscess model of mouse was built against *S. aureus* ATCC25923 or *P. aeruginosa* GIM1.551 as previously described with minor modifications [47]. Twelve groups of 60 mice (5 mice per group) were randomly divided into PMAP-37, PMAP-37(F34-R), Chol-37(F34-R), ampicillin sodium (against *S. aureus* ATCC25923-infected mouse abscess model), benzylpenicillin potassium (against *P. aeruginosa* GIM1.551-infected mouse abscess model), PBS, and blank groups. The fur on the back of each mouse was shaved. Bacterial suspensions (1 × 10^8^ CFU/mL, 50 μL) were injected into the right side of the dorsum underneath the thin skeletal muscle. After 1 h of inoculation, the infected areas were directly injected with PMAP-37, (10 μL, 1 μg/μL), PMAP-37(F34-R) (10 μL, 1 μg/μL), Chol-37(F34-R) (10 μL, 1 μg/μL), antibiotic (ampicillin sodium 10 μL, 1 μg/μL or benzylpenicillin potassium 10 μL, 1 μg/μL), or PBS (10 μL). The treatment program was conducted once per day for 3 days. Mice in the blank group were not challenged and treated. Three days after treatment, mice were euthanized with 120 mg/kg i.p. sodium pentobarbital, and their skin abscesses were harvested and homogenized as described above to assess the bacterial burden.

### Therapeutic analysis of *S. aureus* ATCC25923-infected mouse peritonitis model

The dissemination of *S. aureus* ATCC25923 to target organs was assessed in the peritonitis model. The assessment was performed on the basis of the method described previously with minor modifications [48]. Six groups of 60 mice (10 mice per group) were randomly divided into PMAP-37, PMAP-37(F34-R), Chol-37(F34-R), ampicillin sodium, PBS, and blanks group. Bacterial suspension (1 × 10^8^ CFU/mL, 100 μL) was intraperitoneally injected into mice. After 2 h inoculation, PMAP-37 (20 μL, 1 μg/μL), PMAP-37(F34-R) (20 μL, 1 μg/μL), Chol-37(F34-R) (20 μL, 1 μg/μL), ampicillin sodium (20 μL, 1 μg/μL), or PBS (20 μL) was intraperitoneally injected into the mice, and the treatment program was implemented once per day for 3 days. Mice in the blank group were not challenged and treated. After 3 days of treatment, mice were euthanized with 120 mg/kg i.p. sodium pentobarbital, and the livers and spleens were collected, weighed, and placed in 1 mL PBS. Aliquots of diluted homogenized tissues were spread on LB agar, on which the bacterial burden was determined for analysis. Furthermore, liver and spleen sections were prepared to observe pathological damage as previously reported [49]. The liver and spleen sections were scanned by microscope slide scanner (Pannoramic DESK, 3D HISTECH) and viewed by Caseviewer (C.V 2.3 version). Pathological changes were scored as follows: a score of 1 indicated no pathology, 2 indicated perivascular infiltrates, 3 indicated perivascular and interstitial infiltrates affecting < 20% of the section, 4 indicated perivascular and interstitial infiltrates affecting 20 to 50% of the section, and 5 indicated perivascular and interstitial infiltrates affecting > 50% of the section [50, 51].

### Statistical analysis

The data are presented as means ± standard deviation (SD) of the mean. Statistical analyses were performed using one-way ANOVA F-statistics, and differences were considered significant at *P* < 0.05 or *P* < 0.01.

## Supplementary Information

**Additional file 1: Figure S1.** Antibacterial circle of Chol-37(F34-R). The antibacterial susceptibility testing of Chol-37(F34-R) was performed using the Kirby-Bauer diffusion method on four different bacteria, *S. aureus* ATCC25923 (A), *L. monocytogenes* CICC21634 (B), *S. typhimurium* SL1344 (C), and *P. aeruginosa* GIM1.551 (D). CS, Ceftiofur sodium. The diameter of the standard disk is 6 mm

**Additional file 2: Table S1.** The in vitro antibacterial activity of Chol-37(F34-R) and **Table S2**. Effects of salt ion and serum on the antibacterial activity of Chol-37(F34-R)

## Data Availability

All data included in this study are available upon request to the corresponding author.

## Abbreviations

AMPs: Antimicrobial peptides
DMEM: Dulbecco’s modified eagle medium
DMSO: Dimethyl sulfoxide
LB: Lysogenic broth
BHI: Brain heart infusion
TSB: Tryptic soy broth
MHB: Mueller–Hinton broth
PI: Propidium iodide
MTT: Thiazolyl blue tetrazolium bromide
SPF: Specific pathogen-free
HPLC: High-performance liquid chromatography
MS: Mass spectrometry
MIC: Minimal inhibitory concentration
CFU: Colony–forming units
LPS: Lipopolysaccharide
EPS: Extracellular polymeric substances
SD: Standard deviation
i.p.: Intraperitoneal
