# Supplementary figures and images for: Enhancing the antibacterial activity of antimicrobial peptide PMAP-37(F34-R) by cholesterol modification
Source: BMC Vet Res. 2020 Nov 2;16:419. doi: 10.1186/s12917-020-02630-x (PMC7607875; doi:10.1186/s12917-020-02630-x)

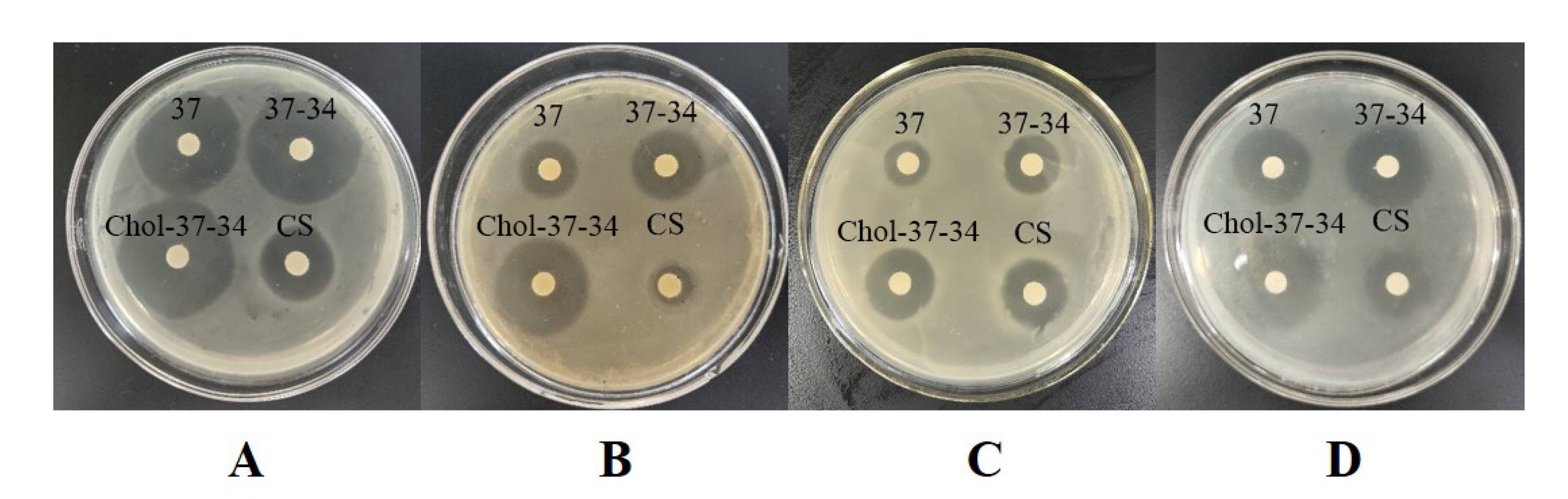

Supplement: Supplementary file 1 — Additional file 1: Figure S1. Antibacterial circle of Chol-37(F34-R). The antibacterial susceptibility testing of Chol-37(F34-R) was performed using the Kirby-Bauer diffusion method on four different bacteria, S. aureus ATCC25923 (A), L. monocytogenes CICC21634 (B), S. typhimurium SL1344 (C), and P. aeruginosa GIM1.551 (D). CS, Ceftiofur sodium. The diameter of the standard disk is 6 mm [file 12917_2020_2630_MOESM1_ESM.tif]
