# Supplementary material for: Enhancing the antibacterial activity of antimicrobial peptide PMAP-37(F34-R) by cholesterol modification
Source: BMC Vet Res. 2020 Nov 2;16:419. doi: 10.1186/s12917-020-02630-x (PMC7607875; doi:10.1186/s12917-020-02630-x)
Supplement: Supplementary file 2 — Additional file 2: Table S1. The in vitro antibacterial activity of Chol-37(F34-R) and Table S2. Effects of salt ion and serum on the antibacterial activity of Chol-37(F34-R) [file 12917_2020_2630_MOESM2_ESM.docx]

**Table S1 The in vitro antibacterial activity of Chol-37(F34-R).**

| Bacteria Strains | PMAP-37 | PMAP-37(F34-R) | Chol-37(F34-R) | ceftiofur sodium |
| --- | --- | --- | --- | --- |
| *S．aureus* ATCC25923 | + | + | +* | + |
| *L. monocytogenes* CICC21634 | + | + | +** | + |
| *S. typhimurium* SL1344 | + | + | +* | + |
| *P. aeruginosa* GIM1.551 | + | + | +** | + |

The vitro antibacterial activity was performed using disk diffusion method. +, peptide have antibacterial activity. *, P < 0.05, and **, P < 0.01 compared with PMAP-37(F34-R).

**Table S2 Effects of salt ion and serum on the antibacterial activity of Chol-37(F34-R).**

| Bacteria Strains | Peptides | | MIC (μg/mL) | | | |
| --- | --- | --- | --- | --- | --- | --- |
|  |  |  | control^a^ | NaCl^b^ | CaCl_2_^c^ | Fetal bovine serum^d^ |
| *S. aureus* ATCC25923 | | PMAP-37 | 0.0313 | 0.0313 | 0.0313 | 0.0313 |
|  |  | PMAP-37(F34-R) | 0.0156 | 0.0156 | 0.0156 | 0.0156 |
|  |  | Chol-37(F34-R) | 0.0078 | 0.0078 | 0.0078 | 0.0078 |
| *P. aeruginosa* GIM1.551 | | PMAP-37 | 2 | 2 | 2 | 2 |
|  |  | PMAP-37(F34-R) | 1 | 1 | 1 | 1 |
|  |  | Chol-37(F34-R) | 0.5 | 0.5 | 0.5 | 0.5 |

The effects of salt ion and serum on antibacterial activities of PMAP-37(F34-R)-C were investigated by MIC assay. a, The control MIC values were determined in the absence of physiological salts and serum. b, c, and d, The final concentrations of NaCl, CaCl_2_, and serum were 8.766 g/L, 0.039 g/L, and 20% (v/v), respectively.
